# Self-Gated Free-Breathing 3D Coronary CINE Imaging with Simultaneous Water and Fat Visualization

**DOI:** 10.1371/journal.pone.0089315

**Published:** 2014-02-20

**Authors:** Jing Liu, Thanh D. Nguyen, Yanchun Zhu, Pascal Spincemaille, Martin R. Prince, Jonathan W. Weinsaft, David Saloner, Yi Wang

**Affiliations:** 1 Department of Radiology, Weill Cornell Medical College, New York, New York, United States of America; 2 Department of Radiology and Biomedical Imaging, University of California San Francisco, San Francisco, California, United States of America; Centre Hospitalier Universitaire Vaudois, Switzerland

## Abstract

The aim of this study was to develop a novel technique for acquiring 3-dimensional (3D) coronary CINE magnetic resonance images with both water and fat visualization during free breathing and without external respiratory or cardiac gating. The implemented multi-echo hybrid 3D radial balanced Steady-State Free Precession (SSFP) sequence has an efficient data acquisition and is robust against motion. The k-space center along the slice encoding direction was repeatedly acquired to derive both respiratory and cardiac self-gating signals without an increase in scan time, enabling a free-breathing acquisition. The multi-echo acquisition allowed image reconstruction with water-fat separation, providing improved visualization of the coronary artery lumen. Ten healthy subjects were imaged successfully at 1.5 T, achieving a spatial resolution of 1.0×1.0×3.0 mm^3^ and scan time of about 5 minutes. The proposed imaging technique provided coronary vessel depiction comparable to that obtained with conventional breath-hold imaging and navigator gated free-breathing imaging.

## Introduction

Coronary magnetic resonance angiography (CMRA) has been highly desired because of its potential clinical significance for managing coronary artery disease [1]. However, the features of the coronary arteries, such as the tortuous shape, the small vessel size, and the sensitivity to motion make routine CMRA still challenging in current clinical practice. To prevent motion artifacts, conventional CMRA acquisitions are usually triggered by the electrocardiographic (ECG) signal and data acquisition is confined to the mid-diastolic period of the cardiac cycle when the heart is relatively at rest. As this period is variable with a changing heart rate, a precise subject-dependent ECG trigger delay needs to be determined. Different segments of the coronary anatomy may also be quiescent at different points in the cardiac cycle [2]. To overcome these problems, the ability to image the coronary arteries throughout the cardiac cycle (coronary CINE imaging) is highly desired.

Effective suppression of the epicardial fat signal is important in CMRA to improve coronary vessel conspicuity [3]. In conventional CMRA, a long spectrally selective RF pulse centered on the fat resonance frequency followed by spoiler gradients is typically used for this purpose. The susceptibility of lung-heart interface can significantly decrease the field homogeneity, causing incomplete fat suppression. Furthermore, such approach interrupts the continuous data acquisition during steady-state, making CINE acquisition difficult. Several alternative fat suppression techniques have been applied to achieve coronary CINE imaging, such as contrast-enhanced or phase-detection techniques [4]–[7]. Effective fat suppression pulses have been applied to coronary CINE imaging by mitigating the steady-state interrupts [8]. Linear combination SSFP (LC-SSFP) method was shown to achieve simultaneous imaging of the coronary lumen and the epicardial fat which is advantageous in enhancing vessel conspicuity at the cost of longer acquisition time [9].

In this study, a novel free-breathing multi-contrast coronary CINE imaging technique is described that provides coronary water and fat CINE images efficiently without requiring dedicated fat suppression pulses. This technique is based on a multiple-echo hybrid 3D radial balanced steady-state free-precession (SSFP) sequence [10], which has been demonstrated to be a promising technique for achieving self-gated free-breathing 3D cardiac CINE imaging for functional measurement and further exploited with water and fat separation in this study. The performance of the proposed technique is evaluated by comparing with the conventional breath-hold CMRA and free-breathing navigator gated imaging techniques in healthy volunteers.

## Methods

This study was approved by the Weill-Cornell Medical Center institutional review board and performed in compliance with the Health Insurance Portability and Accountability Act (HIPAA). Written consent was obtained from all subjects prior to imaging. All imaging experiments were performed on a 1.5T GE HDx scanner (General Electric Healthcare Technologies, Waukesha, WI, USA).

### Self-gated Multi-echo 3D CINE Radial SSFP Sequence

We implemented a multi-echo stack-of-stars 3D balanced SSFP coronary CINE sequence with conventional Cartesian sampling in the k_z_ (slice encoding) direction and radial sampling in the k_x_-k_y_ plane similar to that developed previously for cardiac functional imaging [10]. A unique feature of this sequence is that the acquired k-space center line along the k_z_ direction can provide a direct measure of the global motion of the imaged volume along this direction, from which the superimposed respiratory and cardiac motion can be separated by filtering and used to perform retrospective motion gating of the acquired image data [10]. Unlike conventional ECG-gated coronary imaging sequences, which only capture the coronary arteries in a single cardiac phase (typically during mid-diastole), the proposed sequence acquires data continuously throughout the cardiac cycle and is capable of providing multiple cardiac phase resolved images, from which an image with the optimal depiction of the targeted coronary artery could be selected. A major technical challenge in such continuous data acquisition scheme is effective epicardial fat suppression which is vital for optimal coronary vessel depiction. In this study, the multi-echo data acquisition was exploited to provide simultaneous water and fat images of the coronary arteries, obviating the need for a separate fat suppression pulse.

### Water-fat Separation for Multi-echo 3D CINE Radial CMRA

The multi-echo radial acquisition is capable of separating the water and fat signals by linearly combining data sets acquired at different echo times [11]–[16]. Specifically, the MR signal *d_i_* obtained from a single voxel in the *i*-th echo data set was modeled as follows (ignoring the local static field inhomogeneity) [12]:
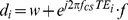
(1)where *w* and *f* denote the water and fat signals respectively, *TE_i_* is the *i*-th echo time, and *f_cs_* denotes the known chemical shift between water and fat peaks (about −210 Hz at 1.5T). The unknowns *w* and *f* can be obtained by inverting the following set of linear equations with a 3-echo acquisition:



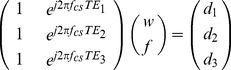
(2)In our study, TEs were chosen such that *TE_2_–TE_1_ = TE_3_–TE_2_* (TEs were 0.3, 1.6, 2.9 ms, respectively).

### Non-linear Combination of Water and Fat Images to Selectively Improve Coronary Lumen Visualization

As described above, we can simultaneously obtain water and fat images of the coronary artery. The water-fat separation usually suppresses the main spectral peak of the fat signal (e.g., −210 Hz at 1.5T) in the water image. However, the other smaller fat spectral peaks may not be well suppressed, which could degrade the contrast between the coronary artery and the surrounding fat [17]. In this study we proposed a simple and fast non-linear combination method of water and fat images to achieve improved coronary lumen imaging as follows:
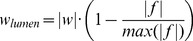
(3)where *w* and *f* are the separated water and fat images respectively, 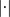 denotes the absolute value (image magnitude), and 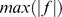 is the manually selected regional maximum intensity of the epicardial fat surrounding the coronary artery. The non-linear image intensity transformation in Eq. 3 scales the water image pixel-wise with a weighting mask derived from the fat image, which has the effect of further suppressing the residual fat signals in mostly fat voxels in the water image where 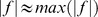.

### Phantom Scans

To demonstrate the water-fat separation and combination techniques in vitro, a water-fat phantom consisting of a balloon filled with peanut oil and immersed into a water container was imaged using the developed multi-echo 3D radial balanced SSFP sequence with the following imaging parameters: TR = 4.9 ms, three TEs = 0.3/1.6/2.9 ms, FA = 40°, BW = ±125 kHz, FOV = 29 cm, spatial resolution = 1.1×1.1×5.0 mm^3^, 16 slices, 400 in-plane projections, scan time of 31 s. The volume body coil was used for signal reception.

### Human Scans

3D CMRA was performed in 10 healthy volunteers (mean age = 29.4 years ±4.2, 9 male, 1 female). An 8-channel cardiac phased array was used for signal reception. The right coronary artery (RCA) and the left anterior descending coronary artery (LAD) were imaged in targeted double-oblique planes in randomized order. The imaging parameters of the self-gated (SG) multi-echo 3D radial balanced SSFP CINE CMRA sequence were as follows: TR = 5 ms, three TEs = 0.3/1.6/2.9 ms, FA = 40°, BW = ±125 kHz, FOV = 26 cm, spatial resolution = 1.0×1.0×3.0 mm^3^, 14–16 slices, free-breathing acquisition without ECG gating. The temporal resolution (the length of each cardiac phase) was 70–80 ms by sharing small amount of high frequency data from adjacent cardiac phases [10], [18]. The scan time was approximately 5 minutes. Retrospective respiratory gating was used to reconstruct CMRA images with a 50% self-gating efficiency.

For comparison, 3D CMRA was also obtained with the conventional breath-hold (BH) ECG-triggered 3D Cartesian balanced SSFP sequence as well as a free-breathing diaphragm navigator (NAV) gated ECG-triggered 3D Cartesian balanced SSFP sequence [19]. The BH sequence was applied with the following imaging parameters: TR = 4.2 ms, TE = 1.7 ms (partial echo), FA = 60°, BW = ±62.5 kHz, FOV = 26 cm, spatial resolution = 1.0×1.6×3.0 mm^3^, 8 slices, 200 ms data acquisition window during the mid-diastolic coronary rest period. The scan time was 24 heartbeats. A subject-specific ECG trigger delay from the R wave to the start of the data acquisition was determined from 2D CINE scout images. The NAV sequence was applied with the following scanning parameters: TR = 4.1 ms, TE = 1.6 ms (partial echo), FA = 60°, BW = ±64 kHz, FOV = 26 cm, spatial resolution = 1.0×1.0×3.0 mm^3^, 14 slices, 128 ms mid-diastolic data acquisition window, same ECG trigger delay as the BH CMRA scan. The phase ordering with automatic window selection [20] real-time navigator gating algorithm was used with a 5 mm gating window. Spectrally selective RF pulses were used for epicardial fat suppression for both BH and NAV CMRA sequences. The three CMRA sequences (SG, BH, and NAV) were performed in a random order for all the subjects.

### Data Analysis

For the water-fat phantom, region-of-interest (ROI) analysis was used to measure the contrast between the water and fat signals. For in vivo CMRA, the contrast between coronary artery (RCA/LAD) and surrounding fat was measured. The coronary ROI was placed on the proximal segment of each coronary artery branch and the fat ROI was placed on the surrounding epicardial fat. The contrast was calculated as 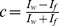, where I_w_ and I_f_ are the average water and fat signal intensities within the ROIs. The change in contrast by applying the water and fat image combination (Eq. 3) was calculated as 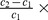
100%, where c_1_ and c_2_ are the measurements without and with the combination, respectively. In this study, we selected the best phase of the cine SG data based on the overall subjective vessel visualization (which considers factors such as coronary sharpness, length, residual motion artifacts and contrast). This optimal cine SG image was used for the measurements in all tables.

Images were reformatted using OsiriX imaging software (OsiriX Foundation, Geneva, Switzerland). For each imaged coronary artery, an experienced radiologist scored the vessel depiction using a 5-point scale: 4-excellent, 3-good, 2-fair, 1-poor, 0-not seen. Wilcoxon paired sample signed-rank test was performed on the scores to assess the difference between the CMRA sequences. A significance level of less than 0.05 was considered to indicate statistical significance. To evaluate the vessel sharpness, the local maximum (*I_max_*) and minimum (*I_min_*) intensity values across the each side of the vessel were determined, from which image sharpness was calculated as the inverse of the distance between 0.8*(I_max_–I_min_)+I_min_* and 0.2*(I_max_–I_min_)+I_min_* [21], [22]. A cross-section of the vessel at the proximal RCA/LAD was chosen for measuring the vessel sharpness. The sharpness obtained at two sides was then averaged for evaluation and comparison. When the vessel sharpness cannot be measured due to excessive motion artifacts, a sharpness of 0 will be assigned. The vessel length of depiction was also measured and evaluated.

The start time, duration and best phase of the rest period were measured as percentages of the cardiac cycle duration for different subjects and coronary branches. Statistical analysis on the measurements (mean and standard derivation) was reported. The measurement difference (p-value) between RCA and LAD was also reported.

## Results

**Figure 1:**
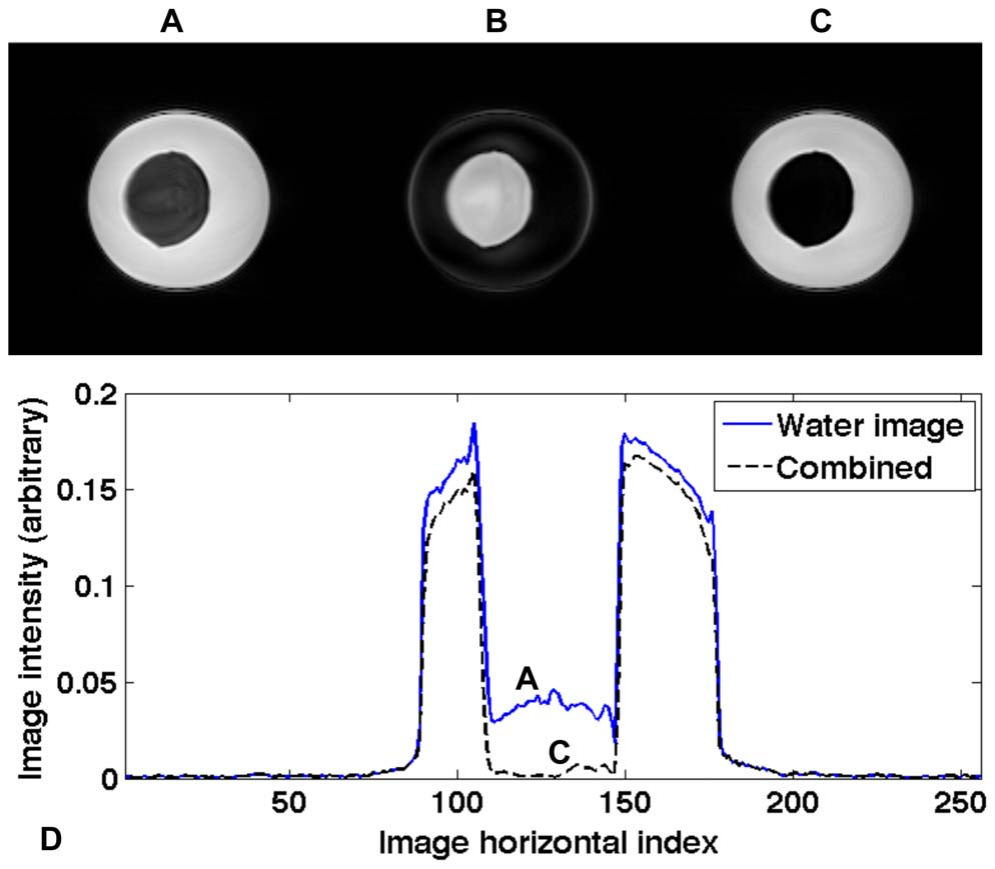
Phantom water image, fat image, and combined image obtained with Eq. 3 are shown in A–C. The central horizontal lines of A and C are plotted in D.

Figure 1A–C shows the separated water and fat images as well as the combined image with Eq. 3 of the water-fat phantom. The combined image (Figure 1C) demonstrates a better contrast (48% improvement) than those in the separated water image (Figure 1A). Figure 1D displays a signal cross-section of the water and combined images (Figure 1A&C), demonstrating the image intensity changes by applying the water-fat combination (Eq. 3).

**Figure 2:**
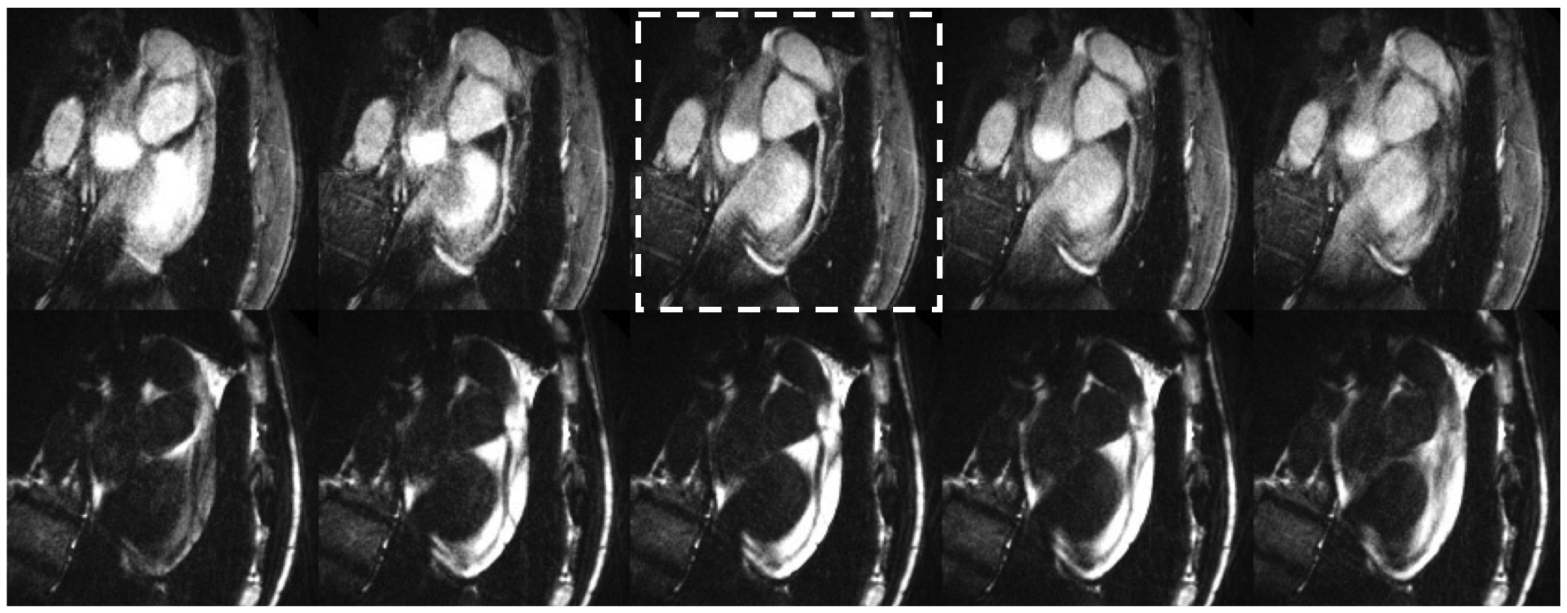
Respiratory and cardiac self-gated free-breathing coronary water (top row) and fat (bottom row) cine images are displayed at five representative phases (every other phase). The right coronary artery is clearly seen in mid-diastole (dashed box) in both water and fat images, and visualization is sensitive to cardiac phase.

CMRA was obtained successfully in all 10 subjects. Figure 2 shows an example of water and fat CINE images of the RCA using the proposed technique at five representative cardiac phases centered around the mid-diastole, demonstrating excellent coronary artery delineation and effective cardiac and respiratory motion suppression in both water and fat images.

**Figure 3:**
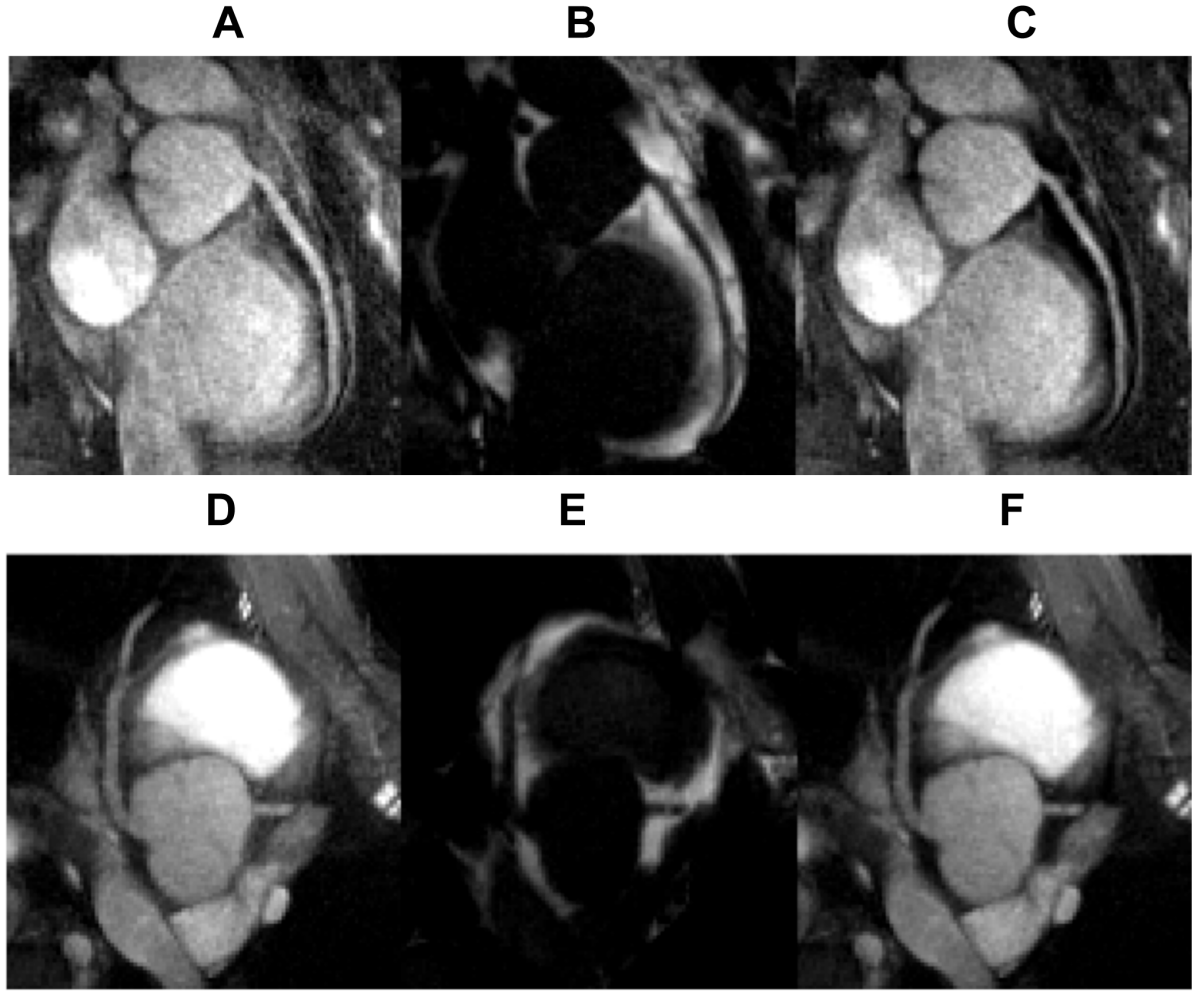
Multi-contrast coronary imaging (top row: RCA, bottom row: LAD). Water images are shown in A&D, fat images in B&E, combined images with enhanced coronary artery contrast (Eq. 3) in C&F.

In Figure 3, water, fat and the combined image with enhanced coronary lumen (Eq. 3) are shown for both RCA and LAD images. Note the improved contrast of the coronary artery lumen against the surrounding fat (Figure 3C).

Table 1 summarizes the coronary artery contrast of RCA and LAD averaged over 10 subjects, demonstrating significantly improved coronary contrast after non-linear combination of water and fat images (Eq. 3), compared to water-only images.

**Table 1:** Coronary artery to epicardial fat contrast measurements and their corresponding changes without and with the non-linear water and fat image combination (Eq. 3) (N = 10).

Artery to fat contrast	Water image	Combined image	Change
RCA	0.7±0.1	1.1±0.3	*52.1% ±38.4%
LAD	0.6±0.7	1.0±0.6	*42.6% ±53.0%

Statistically significant differences (P<0.05) are marked with*.

**Figure 4:**
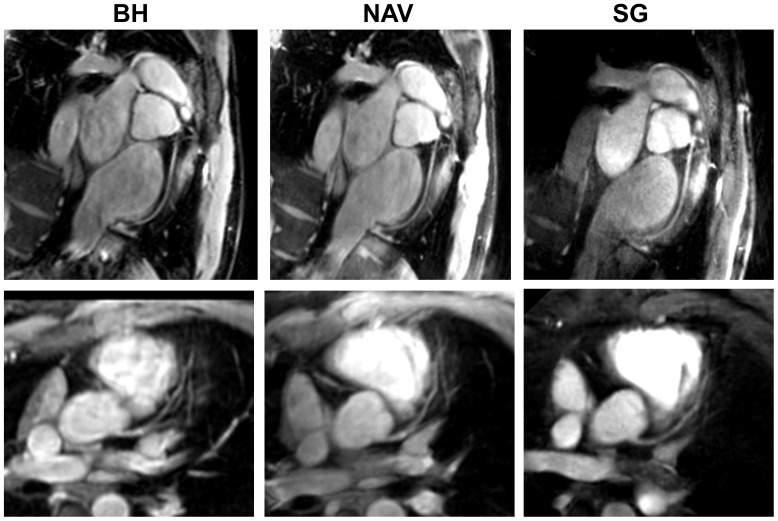
RCA (top row) and LAD (bottom row) images acquired with breath-hold (BH) (left column), free-breathing NAV (NAV) (middle column), and free breathing self-gated (SG) techniques (right column).

Comparisons of BH, free-breathing NAV, and our proposed SG free-breathing techniques are demonstrated in Figure 4. The SG method with water-fat separation generated both water and fat images (only water images are showed as the third column in Figure 4). The NAV scan time was 5.1±1.3 min and the diaphragm navigator gating efficiency was 43±12%. The heart rate was 60.0±6.5 bpm. Both right and left coronary arteries are well depicted. SG method was found to provide slightly lower but statistically comparable (not statistically different) vessel depiction scores when compared to the conventional BH and free-breathing NAV techniques (Table 2).

**Table 2:** Comparison of coronary vessel depiction obtained with breath-hold (BH), free-breathing NAV (NAV), and self-gated (SG) free-breathing CMRA techniques (N = 10, P>0.05).

Vessel depiction	BH	NAV	SG
RCA	3.6±0.7	3.1±1.4	3.0±0.7
LAD	3.4±1.0	3.2±1.1	2.8±0.9

**Figure 5:**
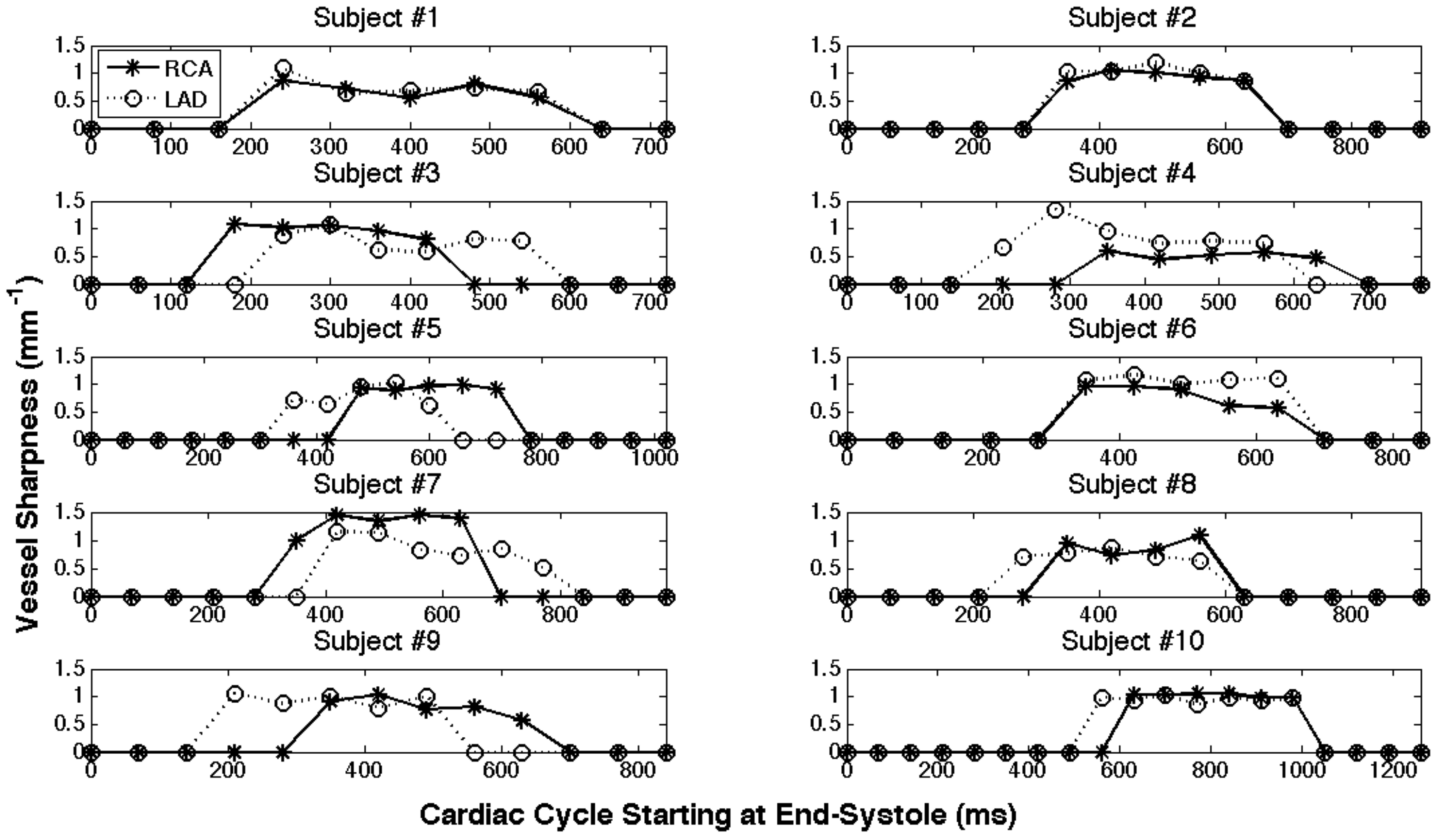
Vessel sharpness obtained with SG technique was measured throughout the cardiac phases. A sharpness of 0 indicates that the vessel sharpness cannot be measured due to excessive motion artifacts.

For the coronary CINE images obtained with SG method, we have measured vessel sharpness of both RCA and LAD during all cardiac phases where the vessels were not corrupted by motion (referred as rest period). The sharpness values at cardiac phases where the vessel was corrupted by motion were assigned with zeros. Figure 5 shows the change in coronary vessel sharpness throughout the cardiac cycle for all 10 subjects. Note that the cardiac phases start at end-systole. The start time, duration and best phase of the rest period vary for different subjects and coronaries. The statistical analysis of the vessel rest period is summarized in Table 3. It shows that the LAD rest period starts earlier and lasts longer than that of RCA. RCA rest period has a large variation among subjects.

**Table 3:** Statistical analysis of RCA and LAD rest period.

% of thecardiac cycle	Best Phase location	Start Time	Rest Duration
RCA	54.7±19.0	44.1±6.4	35.9±7.2
LAD	48.6±9.5	40.3±6.2	39.6±7.2
RCA v.s. LAD	P = 0.19	*P = 0.02	P = 0.38

All numbers are report as percentages of the cardiac cycle duration. The difference between the measurements of RCA and LAD is marked with *, if the difference is statistically significant (P<0.05).

Vessel sharpness and length of depiction were measured for BH, NAV and SG methods. For SG method, only the best cardiac phase was analyzed. The results are summarized in Table 4, showing similar performance among the three techniques (all P values ≥ 0.05, N = 10).

**Table 4:** Comparison of coronary vessel length of depiction and sharpness obtained with breath-hold (BH), free-breathing NAV (NAV), and self-gated (SG) free-breathing CMRA techniques (N = 10).

Measurement		BH	NAV	SG
Length (cm)	RCA	12.6±2.2	13.2±1.6	11.5±2.3
	LAD	10.5±2.5	9.6±2.5	10.2±1.3
Sharpness (mm^−1^)	RCA	0.92±0.26	1.00±0.10	0.93±0.15
	LAD	0.87±0.15	1.05±0.24	1.01±0.14

The p-values between methods are great than 0.05 (the difference is not statistically significant).

## Discussion and Conclusion

It is well known that there exists a large variability in starting point and duration of the coronary rest period (depending on subject and coronary branches) with no correlation to heart rate [2]. Conventional single-cardiac-phase coronary imaging would ideally require the precise estimation of patient- and vessel branch-specific trigger delay and acquisition window, which is difficult and can be time-consuming. Our preliminary results have demonstrated the feasibility of simultaneous water and fat 3D CINE imaging of the coronary arteries during free breathing without the need for external motion gating devices. Given the segment-to-segment variation in coronary motion through the cardiac cycle, CINE imaging permits optimal retrospective selection of the cardiac phase that provides the best visualization for any given coronary segment, a major benefit over existing single cardiac phase CMRA techniques. Future studies in a larger population will be needed to demonstrate this potential advantage of the cine imaging approach.

The self-gating method applied in this study has been validated for cardiac CINE imaging for functional measurement, by comparing to the standard external measures [10], [23]. Although the orientation of the targeted coronary artery imaging might be different from that of the cardiac CINE imaging, we have observed reliable gating signals in our coronary imaging studies. Respiratory motion of the heart is mainly along the head-foot direction. Although the motion projecting to k_z_ direction of an arbitrary oblique plane may have an under-estimated amplitude, the respiratory motion waveforms (motion frequencies) remain. Thus the self-gating signal based on the center-of-mass can still be derived with a band-pass filtering. In this study, a fixed respiratory gating efficiency 50% was applied [10], however a subject-specific gating efficiency needs to be further investigated.

In this study, the coronary image quality was found to be similar for the proposed SG technique and the conventional BH and NAV techniques, although these sequences had different temporal resolutions of 78 ms, 200 ms and 128 ms, respectively. This may be explained by the relatively lower heart rate (correspondingly longer mid-diastolic cardiac rest period) and the ability to sustain a long breath-hold (24 heartbeats) in young healthy volunteers of this study. Comparison of image quality in a patient population will be considered in our future work.

Arrhythmia was not observed in the healthy volunteers recruited for this study. Heart rate has a direct effect on the acquisition time of the breath-hold sequence and together with respiratory motion influences the acquisition time of the diaphragm navigator gated sequence. The acquisition time of the BH sequence changed according to the heart rate, since a fixed number (24) of heart beats were acquired. We did not observe a significant correlation between the heart rate and scan time for the navigator-gated sequence. The acquisition time of the self-gated radial sequence was fixed at 5 minutes.

The quality of the coronary MRA is primarily determined by the effectiveness of motion suppression (both cardiac and respiratory) and to a lesser degree by the spatial resolution. The higher quality of the breath-hold sequence as observed in this study, while surprising in light of previous studies [24], can be explained by our special study population comprising of young, healthy and seasoned volunteers who have low heart rates (hence minimizing cardiac motion effects) and are capable of sustaining a long breath-holds of 24 heartbeats. The images were reviewed by a radiologist with five years experience in cardiovascular MRI. As our study population consists of young seasoned volunteers capable of sustaining good-quality long breath-holds, the reader gave higher scores to breath-hold images which generally have less respiratory motion artifacts when compared to the navigator gated images. We expect a different result in an older patient population, which will be studied in subsequent works.

The current temporal resolution (∼70–80 ms) with the proposed SG technique is not high enough to provide nice coronary images throughout all the cardiac phases (Figures 2&5). But with radial acquisition and view sharing reconstruction, the SG technique could provide reasonable coronary images at multiple cardiac phases rather than one or two using conventional methods (BH or NAV). Since the self-gating method is based on the blood intensity change during the cardiac cycle, the cardiac phases showed in Figure 5 start at end-systole (with the smallest amount of blood signal) [10]. Table 3 shows that the LAD rest period starts earlier and lasts longer than that of RCA, which agrees with what was reported earlier [2]. The large variance of RCA rest period (±19% of the cardiac cycle) among different subjects indicates that patient-dependent trigger delay is crucial for the conventional approach using an ECG-triggered single-phase imaging sequence, while it is much less problematic for the multi-phase cine sequence developed in this work.

A simple model of water-fat separation without considering the local field inhomogeneities was used in this study. When used at 1.5 T field strength in combination with targeted volume shimming to reduce field inhomogeneities in the heart region, our technique was shown to consistently provide robust epicardial fat suppression and coronary angiograms of good quality in our study cohort comprised of young healthy volunteers. However, water-fat separation with simple Dixon method can be problematic when good shimming is difficult to achieve (e.g., in larger patients), and a more advanced Dixon method may be needed. The comparison of our technique and more advanced water-fat separation methods that take into account local inhomogeneity, such as in generalized three-point Dixon, IDEAL, or VARPRO methods [12], [13], [16], will be considered in our future work.

As reported in Tables 1&4, the decreased values of the SG method are not statistically significant compared to the other two methods. However, we have to note that selecting the best rest period of the cardiac cycle with the proposed method may not lead to the best image quality, which may also be affected by many other aspects, such as respiratory motion, sampling trajectory, data undersampling, fat-sat strategy, and so on. Although a fairly large 50% gating window was intentionally chosen in this study to keep the acquisition time for SG and NAV methods similar, a narrower gating window (20∼30%) may be used to improve coronary quality if more scan time is available. The Dixon method used in our study was based on multi-echo radial acquisition. Although we had corrections for gradient delays and eddy currents, the residual errors may still affect the radial trajectories within a single echo data set or among different echo data sets and result in image quality degradation. Thus, a different fat-sat strategy used with respect to BH and NAV could also influence the vessel sharpness and the subjective score.

In this study, a nonlinear filtering was used to enhance the contrast of the coronary artery by combining the simultaneously obtained water and fat images (Eq. 3). A region of epicardial fat surrounding the coronary artery was manually selected and its averaged intensity was calculated as the regional maximum intensity for Eq. 3. Although this value is just an estimation of the fat signal intensities around the vessel, this nonlinear filtering method is relatively insensitive to the inhomogeneity of the fat signal, especially compared to a simple linear mask based on a threshold of the fat signal (data not shown). Because of the nonlinear filtering, the background noise of the combined images has complicated statistics and cannot be used to derive noise power in the real/imaginary channel. Therefore, in this study we only evaluated the relative contrast between the water and fat signals, but did not report signal-to-noise ratio (SNR) or contrast-to-noise ratio (CNR) measurements. We have also done numerical simulations to evaluate the contrast improvement effect (Eq. 3). Reliable contrast improvement has been achieved with different variations of the water and fat signal intensities (data not shown).

In this study, the conventional targeted-volume approach was used for coronary imaging with a scan time of 5 minutes per main coronary artery branch. Our experience as well as more recent studies [25], [26] have indicated that targeted-volume imaging generally provides better coronary image quality than whole-heart imaging, although the latter approach provides much simplified planning of scan planes, potentially resulting in shorter total scan time, and may prove useful in visualizing smaller coronary side-branches [27]. While our free-breathing radial coronary sequence can be applied directly to whole-heart imaging, advanced acceleration methods such as parallel imaging [28]–[31] and compressed sensing [32] are being investigated to reduce the whole-heart acquisition time to within the clinically useful limit of approximately 5–10 min. Use of intravascular contrast agents to boost blood SNR may also help improve image quality or shorten scan time. To further improve data efficiency and reduce residual motion artifacts, the respiratory self-gating can be exploited for motion correction [33]–[36]. The idea of using repeatedly acquired radial lines to derive the self-gating signals and further compensate the motion in [34] would be suitable for our approach. Based on the similar idea, we could derive 3D motion information for correction, as demonstrated in our preliminary study of cardiac CINE imaging [37]. Its application to coronary CINE imaging will be further explored.

In conclusion, the proposed self-gated coronary CINE imaging can simultaneously provide cardiac phase resolved water and fat images of the coronary arteries.
